# Highly selective tungstate transporter protein TupA from *Desulfovibrio alaskensis G20*

**DOI:** 10.1038/s41598-017-06133-y

**Published:** 2017-07-19

**Authors:** Ana Rita Otrelo-Cardoso, Rashmi R. Nair, Márcia A. S. Correia, Raquel S. Correia Cordeiro, Alejandro Panjkovich, Dmitri I. Svergun, Teresa Santos-Silva, Maria G. Rivas

**Affiliations:** 1 UCIBIO/REQUIMTE, Departamento de Química, Faculdade de Ciências e Tecnologia, Universidade Nova de Lisboa, 2829-516 Caparica, Portugal; 2 European Molecular Biology Laboratory-Hamburg Outstation, c/o DESY, Notkestrasse 85, D-22607 Hamburg, Germany; 3 Department of Physics, Facultad de Bioquímica y Ciencias Biológicas, Universidad Nacional del Litoral, Santa Fe, 3000 Argentina; 4 Ruhr-Universität Bochum, Universitätsstraße, 150/44780 Bochum, Germany

## Abstract

Molybdenum and tungsten are taken up by bacteria and archaea as their soluble oxyanions through high affinity transport systems belonging to the ATP-binding cassette (ABC) transporters. The component A (ModA/TupA) of these transporters is the first selection gate from which the cell differentiates between MoO_4_^2−^, WO_4_^2−^ and other similar oxyanions. We report the biochemical characterization and the crystal structure of the apo-TupA from *Desulfovibrio desulfuricans* G20, at 1.4 Å resolution. Small Angle X-ray Scattering data suggests that the protein adopts a closed and more stable conformation upon ion binding. The role of the arginine 118 in the selectivity of the oxyanion was also investigated and three mutants were constructed: R118K, R118E and R118Q. Isothermal titration calorimetry clearly shows the relevance of this residue for metal discrimination and oxyanion binding. In this sense, the three variants lost the ability to coordinate molybdate and the R118K mutant keeps an extremely high affinity for tungstate. These results contribute to an understanding of the metal-protein interaction, making it a suitable candidate for a recognition element of a biosensor for tungsten detection.

## Introduction

Tungsten is a rare transition metal element from the group VI of the periodic table, together with chromium and molybdenum. It presents several important characteristics that include a high melting point (the highest of all metals, 3422 °C), good conductivity and a large variety of oxidation states (from −2 to + 6)^1, 2^. Due to its versatility, W is an important component of a broad range of civil and military industries. The applications include X-ray equipment, implanted medical devices, microwaves, ammunitions, light bulb filaments, jewelry, metal tools, among others^3, 4^. Since the end of the 19th century, Portugal has an important W deposit, being the ninth major producer in the world (by the U.S. Geological Survey in 2016)^5^. In the past, W was viewed as a non-toxic metal but the scientific community became aware of its potential impact on the environment, through the accumulation in air, soils and water (where WO_4_^2−^ is the dominant species), mainly in mining sites, but also in some industrial and military sites. Recent data indicate that high concentrations of W are associated with peripheral arterial disease^6^, increased prevalence of stroke^7^, fasting plasma glucose levels and diabetes^8, 9^.

In biological systems, the incorporation of tungsten as a cofactor is limited to bacteria and archaea, with the W-containing enzymes almost restricted to obligate anaerobic prokaryotes^10, 11^. In contrast, the similar element molybdenum can be also found in several enzymes from Eukarya. In most cases, the function of Mo and W-enzymes is to catalyze oxygen atom transfer reactions. One of the challenges of biological systems is to differentiate one metal from the other to avoid its incorrect insertion in the catalytic site of enzymes. Misincorporation of metals into proteins often leads to compromised activity or even inactive enzymes^12–14^.

Both tungsten and molybdenum are taken up by cells in the form of their soluble oxyanions (MoO_4_^2−^ or WO_4_^2−^) by high specific ABC transporters^15, 16^. In prokaryotes, there are three types of tungstate/molybdate transporters: ModABC^17^, TupABC^18^ and WtpABC^19^. They are composed of a periplasmic protein (component A), a transmembrane pore forming protein (component B) and a cytoplasmic protein (component C), which hydrolyzes ATP to transport the oxyanion into the cell cytoplasm^16^. The genes encoding the three components are organized in an operon (*mod*/*wtpABC*) or gene cluster and regulated by a transcription factor known as ModE (in the case of the ModABC operon)^20^. The *modA*/*tupA*/*wtpA* gene encodes a periplasmic molybdate/tungstate-binding protein (protein A) which constitutes the first selection gate from which cells should differentiate between Mo and W. The ModA, WtpA, and TupA proteins differ in their binding affinity for molybdate versus tungstate, primary sequence and oxoanion coordination chemistry^16, 18, 19, 21–23^. The dissociation constants reported to date suggest that TupA and WtpA are strongly selective for tungstate whereas ModA cannot discriminate between molybdate and tungstate^16^.

Several crystal structures of ModA/WtpA proteins are available in Protein Data Bank (PDB), namely Mod/WtpA from *Pyrococcus furiosus* ^24^ and ModA from *Escherichia coli* ^25^. In contrast, TupA has been poorly studied. Despite the existence of the TupA crystal structure of *Geobacter sulfurreducens* (*Gs*TupA) deposited in the Protein Data Bank (PDB code 3lr1), this system has only been characterized in *Eubacterium acidaminophilum* ^18^ and *Campylobacter jejuni* ^26^. Atomic detail of tungstate-binding proteins is crucial to understand ligand coordination and the mechanism of metal differentiation, mainly if biotechnological applications are considered.

The present work reports the 1.40 Å crystal structure of the recombinant apo-form of TupA protein from the sulfate reducing bacteria *Desulfovibro alaskensis* G20 (*Da*G20 TupA) together with the biochemical characterization of three variants: R118K, R118E and R118Q. The Arg118 substitution by a lysine yields a variant that is not able to bind molybdate but keeps an extremely high affinity for tungstate. Small-angle X-ray scattering (SAXS) and X-Ray data of the recombinant protein provides valuable information about structural characteristics of TupA and residues involved in ion binding. The last, together with the unique properties of the R118K mutant, contribute for the development of highly selective tungstate sensors.

## Results and Discussion

### Overall structure

**Figure 1:**
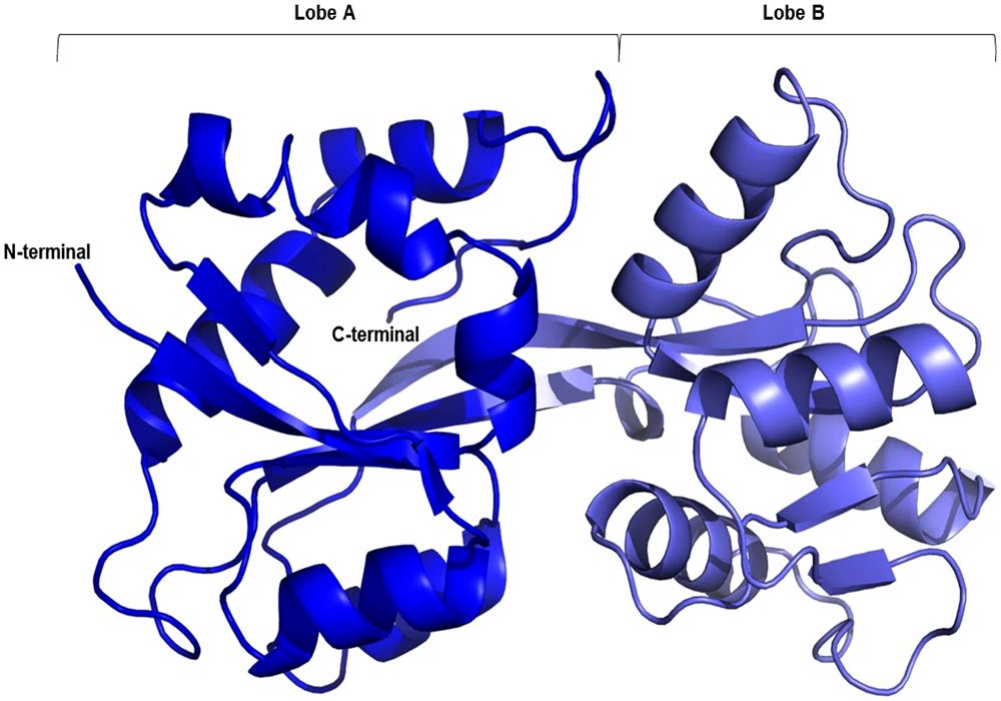
Cartoon representation of the *DaG2*0 TupA tertiary structure. The different colors represent the two lobes, A in blue and B in light blue. Picture prepared with Pymol^50^.

TupA is a globular protein with approximate dimensions of 55.7 × 34.5 × 23.8 Å (Fig. 1). The protein has a butterfly shape with a central beta strand (T77-P89) that supports two lobes (lobe A: A2-R76 and Y201-E251; lobe B: A91-Q200). This architecture was first described for ModA from *E. coli* ^25^ and later for ModA from *Archaeoglobus fulgidus* ^27^. The central beta strand is hydrogen bonded to three beta strands of each of the lobes forming two 4-stranded beta sheets. The TupA metal binding site is located in a central cleft assembled by the two lobes. Despite the very low sequence identity between lobes A and B (5.2%), their secondary structure elements and overall fold is very similar. In fact, superposition of the two lobes gives a RMSD of 3.0 Å (for 57 Cα) showing high resemblance between the two parts of the protein that hold the metal ion. This could be associated with the fact that *Da*G20 TupA is a monomeric protein suspected to interact with dimeric components of the ABC transporting system. The structural similarities observed between the two lobes could be a result of an evolutionary strategy adopted to improve the interaction between the monomeric and dimeric membrane associated components. This structural characteristic extends to other members of the TupABC family such as *Gs*TupA (UniProt Q749P2, PDB code 3lr1) (superposition of the two lobes gives a RMSD of 3.1 Å (for 37 Cα), but also to ModABC transporting family (*eg* ModA from *E. coli* ^25^, where a RMSD of 2.92 Å is obtained for superposition of 63 Cα) and WtpABC (*eg* ModA/WtpA from *Pyrococcus horikoshii* ^24^ with a RMSD of 3.05 Å from 65 Cα).

### Comparison of *Da*G20 TupA with related structures

**Figure 2:**
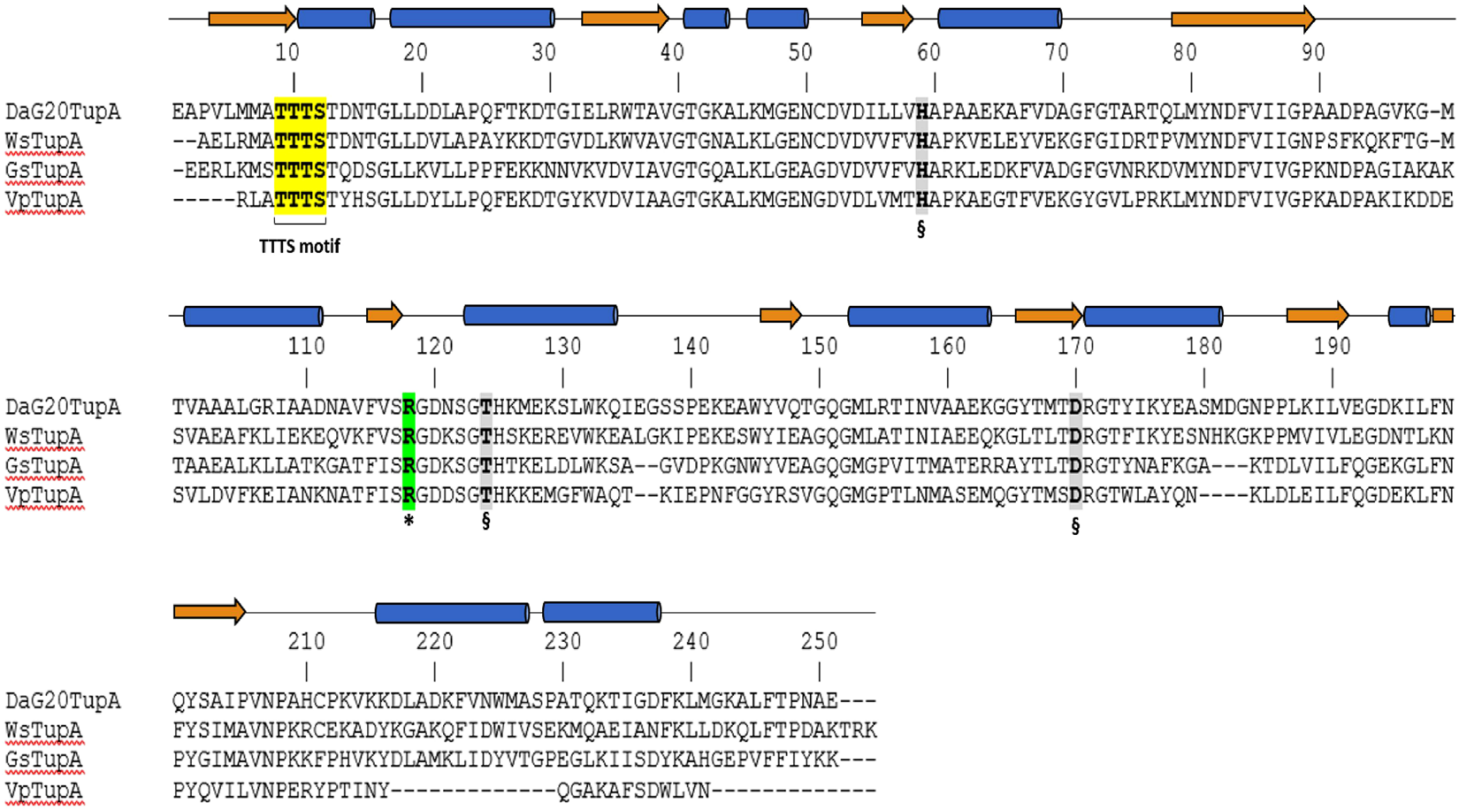
Multiple sequence alignment of mature TupA proteins from different organisms. DaG20TupA - TupA from *Desulfovibrio alaskensis* G20 (locus tag Dde_0234); WsTupA - TupA from *Wolinella succinogenes* (locus tag WS1370*)*; GsTupA - TupA from *Geobacter sulfurreducens* (locus tag GSU2700) and VsTupA - TupA from *Vibrio parahaemolyticus* (locus tag VP1501). Important (*) and putative (^§^) residues involved in oxyanion binding.

There are several models available at the PDB of molybdate/tungstate binding proteins. The ModA proteins from eubacteria or archaea share low sequence homology with *DaG20* TupA (less than 22%) and, even though the proteins fold is similar, the RMSD upon superposition is over 3 Å. When the 3D model reported here is used as a search model at the PDB, three bacterial proteins with over 45% sequence identity are retrieved. These are the already mentioned *Gs*TupA a protein with unknown function from *Vibrio parahaemolyticus* (Q87PK2, PDB code 3muq) and a LysR substrate binding domain from *Wolinella succinogenes* (Q7M8V9, PDB code 3kn3), all obtained by the Protein Structure Initiative (PSI). In common, the proteins contain the TTTS motif and some other important residues for tungstate binding (see below), indicating that they should be classified as the component A of the TupABC system (Fig. 2).

The *Da*G20 TupA shares 45.6% sequence identity with *Gs*TupA, with conserved secondary structure elements, suggesting that the two proteins are structurally very similar. However, superposition of the entire protein provides an unexpectedly high RMSD value (2.4 Å upon superposition of 208 Cα). A more realistic comparison can be achieved when considering the superposition of the independent lobes of the two proteins, yielding much lower deviations (RMSD for the superposition of lobes A and B from the two proteins is 1.24 Å and 1.16 Å for 106 and 96 Cα, respectively). These values support a high structural similarity between *Da*G20 TupA and *Gs*TupA but also indicate some degree of flexibility of the lobes with respect to one another, as expected for the periplasmic A proteins of these transporters. *Gs*TupA was likely crystallized in the presence of an ionic form of tungsten, and the deposited model reports a free W^6+^ accommodated in the metal binding cleft. In this *Gs*TupA-W^6+^ holo form, the central cleft volume (363.9 Å^3^) is four times smaller than the apo form of *Da*G20 TupA here reported (1480 Å^3^). Unexpectedly, the cation is not coordinated to water molecules or protein residues. The water molecules surrounding the W^6+^ are at a distance of 3.23–3.82 Å and the closest residues are at 3.9 Å (the OG1 of T9 and NH1 of R118 (*Gs*TupA numbering) from lobes A and B, respectively). In *Da*G20 TupA these residues are at the same position although not superimposable with *Gs*TupA. The data suggest that conformational changes take place upon metal binding, where the protein in the holo form adopts a more compact conformation.

### Analysis of the overall structure and stability upon ligand binding

To elucidate the possible conformational changes of *Da*G20 TupA upon ligand binding, synchrotron SAXS data were collected both in the presence and absence of tungstate and molybdate. The scattering data of the apo form indicate a monomeric globular TupA protein with a radius of gyration (*R*_*g*_) of 24.2 Å and a maximum particle size (*D*_*max*_) of about 95 Å (see Table 1). In the presence of tungstate or molybdate (datasets *Da*G20 TupA-WO_4_^2−^ and *Da*G20 TupA-MoO_4_^2−^, respectively), the overall shape of the protein remains globular but becomes more spherical and compact, leading to a decreasing *R*_*g*_  (23 Å) and *D*_*max*_  (90) Å. Importantly, the datasets obtained for TupA in the presence of tungstate and molybdate are very similar: the CorMap approach^28^ reveals no statistically significant difference (C = 12, *p*-value = 0.12) between both datasets, indicating that in solution the protein adopts the same conformation upon binding both oxyanions.

**Table 1:** Data collection and structural parameters obtained by SAXS.

	TupA	TupA*	TupA W	TupA W*	TupA Mo
Data collection parameters					
Instrument	P12	BM29	P12	BM29	P12
Wavelength, Å	1.24	0.99	1.24	0.99	1.24
s-range, Å^−1^	0.0027–0.45	0.003–0.49	0.0027–0.45	0.003–0.49	0.0027–0.45
Exposure time, s	1	10	1	10	1
Concentration range, mg/ml	1.0–8.5	0.8–6.0	1.0–6.0	0.8–12.0	1.0–6.0
Temperature, K	280	278	280	278	280
Structural parameters					
R_g_, Å [from *P(r)*]	25.5 ± 0.3	25.4 ± 0.3	24.1 ± 0.2	23.8 ± 0.2	24.4 ± 0.2
R_g_, Å (from Guinier)	24.2 ± 0.4	24.0 ± 0.4	23.0 ± 0.3	22.8 ± 0.3	22.9 ± 0.3
D_max_, Å	96 ± 3	95 ± 3	90 ± 3	89 ± 3	92 ± 3
Porod volume, Å^3^ × 10^3^	51.4 ± 3.5	48.5 ± 3.3	45.7 ± 3.1	44.4 ± 3.0	45.8 ± 3.1
Mol. mass determination (kDa)					
From *I(0)*	29 ± 2	32 ± 2	26 ± 2	29 ± 2	26 ± 2
From Porod volume	31 ± 2	29 ± 2	28 ± 2	27 ± 2	28 ± 2
Model vs. data discrepancy (χ^2^)					
Holo-form model	4.0	2.4	**0.9**	**2.2**	**0.9**
MX	7.8	5.8	1.8	11.6	1.8
Apo-form model	**1.0**	**0.7**	2.0	11.9	1.7

Molecular mass (M) was estimated from forward scattering I(0) and Porod volume respectively, the theoretical molecular mass predicted from sequence is 29.7 kDa. Radius of gyration, *R*_*g*_ (Å) was calculated using the Guinier approximation and also the distance distribution function (*P*(*r*) using GNOM), which also estimates maximum particle dimension (*D*_*max*_). χ^2^ values correspond to discrepancies between models and experimental data, the lowest χ^2^ value per dataset is highlighted. MX: crystal structure.

*Data collected at BM29 beamline, ESRF, Grenoble, France. The rest of the datasets were collected at EMBL P12 beamline, DESY, Hamburg, Germany.

**Figure 3:**
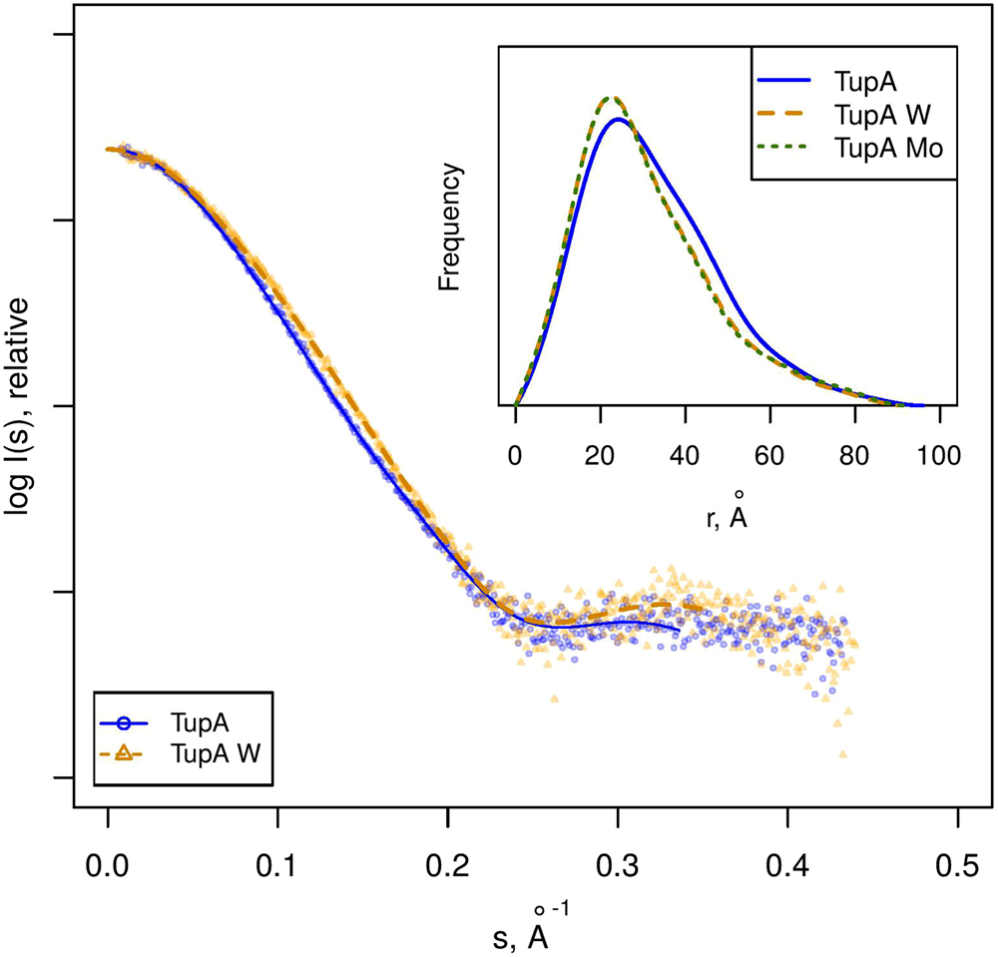
SAXS scattering data (points) and GNOM fits (lines) for TupA in the absence (TupA) and presence of tungstate (TupA W). The data collected in presence of molybdate was omitted from the main plot for clarity as it matches the TupA W data up to noise. Insert: distance distribution functions (*P*(*r*)) for the different conditions measured.

Comparison of the distance distribution functions (*P*(*r*)) between holo and apo forms shows a more compact conformation for the holo form, in agreement with the idea that the protein ‘closes’ upon binding of the metal ion. The apo-form *P*(*r*) shape supports the notion of the two lobes being more separated than in the holo-state. The slowly decaying long interdistance tail observed in the *P*(*r*) function (Fig. 3, insert), suggests an unstructured N-terminal region containing the 23 residues long expression-tag.

**Figure 4:**
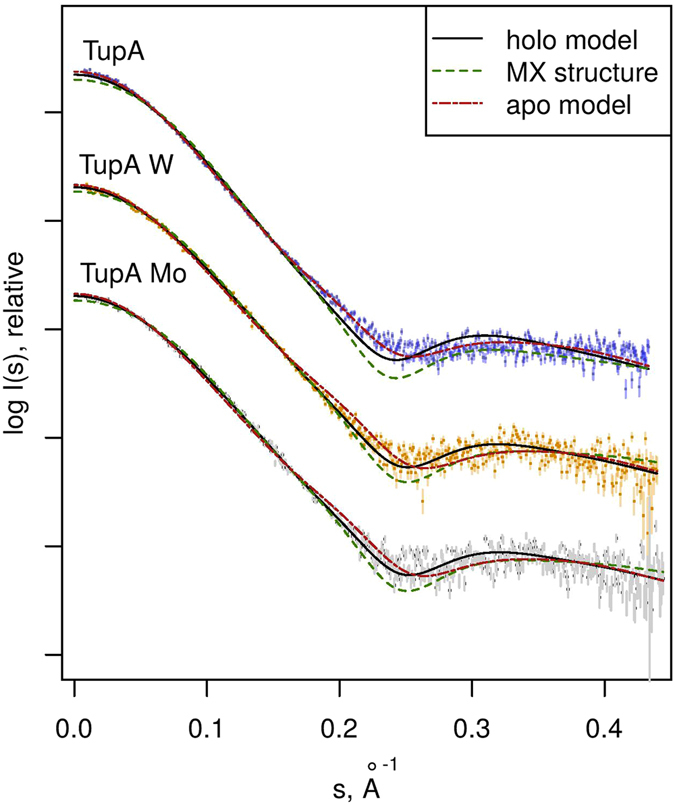
SAXS scattering data (points) for the three experimental conditions, TupA in the absence (TupA) and presence of tungstate (TupA W) or molybdate (TupA Mo). Each dataset has been scaled for display purposes. For each experimental curve, CRYSOL fits are displayed for the crystallographic structure reported in this work (MX) and the holo- and apo-form models generated thereof. The best fit for TupA is the apo-form model (χ^2^ = 1.0), while the best fit for both TupA W and TupA Mo is the holo-form model (χ^2^ = 0.9).

**Figure 5:**
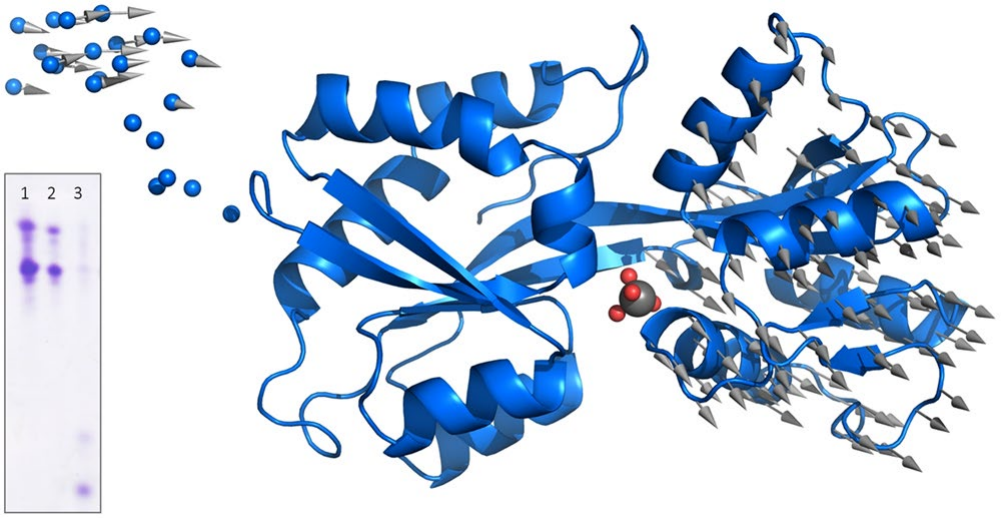
Three-dimensional coordinates for the holo-form hybrid model are displayed using a cartoon representation. The N-terminal section modeled with BUNCH is shown as small spheres. The large gray sphere in the center corresponds to the tungstate group (small red spheres represent O atoms) modeled by homology with PDB entry 3cfz and 3lr1. Vectors have been drawn connecting C^α^s from the holo-form model to the apo-form model generated by SREFLEX, after superposition of lobe A, to display the ‘opening’ conformational transition. Upon optimal superposition including all C^α^ (274), the RMSD is 1.5 Å. Insert: Urea-polyacrylamide gel electrophoresis of (1) TupA, (2) TupA+MoO_4_, (3) TupA+WO_4_. The samples in presence of ligand were first passed through a size exclusion PD-10 minitrap G-25 columns to eliminate the excess.

The crystal structure reported here was used as a starting point to create models for both the holo-form and apo-form states. The 23 N-terminal residues that are missing in the crystal structure were calculated using BUNCH^29^. Based on *Gs*TupA-W^6+^ adduct, a tungstate group was added to the expected binding site of the BUNCH model, generating a theoretical holo-form model. The theoretical scattering curves calculated using this holo-form model of *Da*G20 TupA are in very good agreement with the SAXS measurements of the protein in the presence of tungstate or molybdate (discrepancy χ^2^ = 0.9) (Fig. 4). The apo-form model was created by refining the initial BUNCH model with the SREFLEX program^30^. This approach revealed a slight opening of the lobes (RMSD of 1.5 Å for 274 Cα atoms) yielding an excellent agreement to the TupA SAXS data measured in the absence of tungstate or molybdate (χ^2^ = 1.0). The optimum holo-form model yielding the best χ^2^ to the experimental data and the smallest RMSD to the original structure is presented in Fig. 5 and the conformational transition holo-apo is depicted by arrows. The SAXS data corroborate the hypothesis that *DaG20* TupA is a flexible protein that is able to adopt a loose conformation in the free form but, when binding to molybdate or tungstate, switches to a more compact fold. This feature can be explored to design new detection systems, as discussed later.

To study the impact of the metal binding in the protein stability urea-polyacrylamide gel electrophoresis was carried out for the recombinant *DaG20* TupA in the presence/absence of tungstate and molybdate. TupA-ligand samples were prepared using a 10-fold excess of metal and the excess was removed by size exclusion chromatography. The gel shows that, upon tungstate binding, the wild-type protein migrates in the gel in a larger extend than in the absence of metal or in the presence of molybdate. This indicates that TupA adopts a more compact conformation that is likely to increase stability under a urea gradient, in agreement with our SAXS analysis (Fig. 5, insert).

### Oxyanion binding site

**Figure 6:**
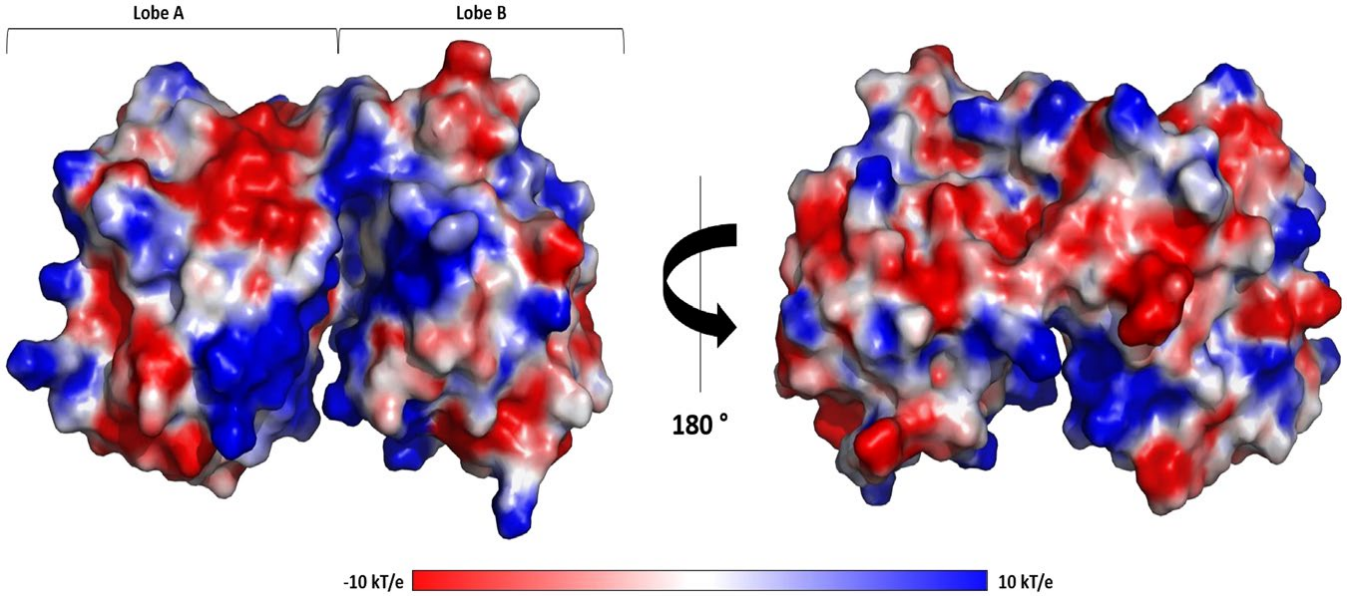
Electrostatic potentials of TupA surface. Electrostatic surface potentials were calculated using Pymol^50^. Surface potentials varies from −10.0 kT/e (red) to 10 kT.0 kT/e (blue).

**Figure 7:**
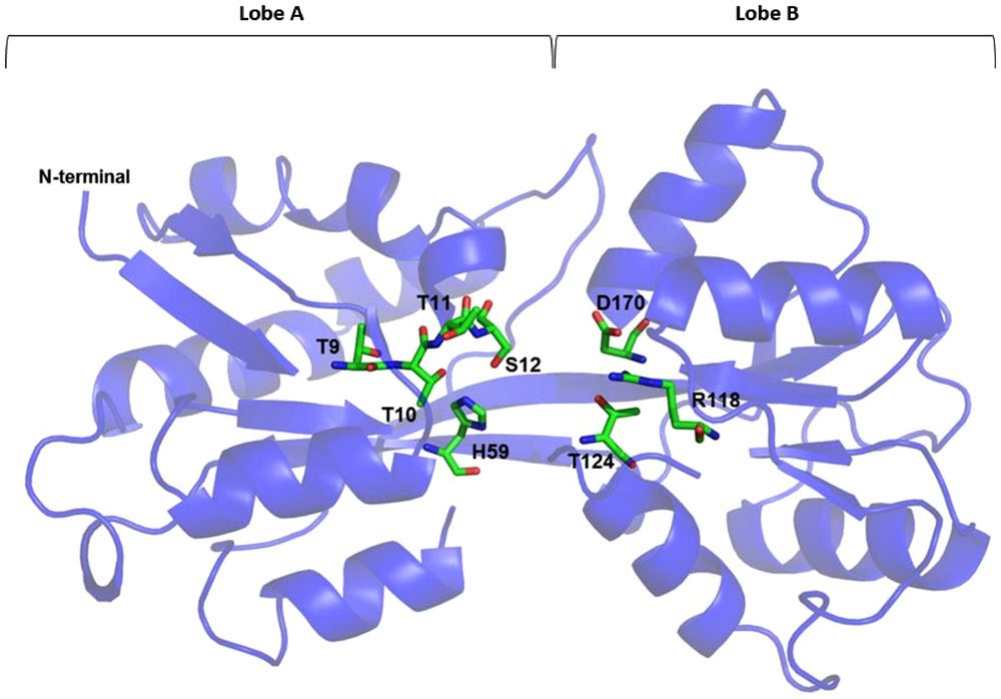
Cartoon representation of the *DaG2*0 TupA tertiary structure with the conserved residues involved in the metal binding site highlighted. The His59, Thr9-11, Ser12, Arg118, Thr124 and Asp170 are represented as sticks and color by element. Picture prepared with Pymol^50^.

Most of the residues that form the binding cleft of ModA are essentially polar but poorly conserved among proteins from different organisms, as discussed for *Ec*ModA^25^. In *DaG20* TupA, the cleft is decorated with positively charged residues as seen by the electrostatic surface potential calculations (Fig. 6). The pronounced positive environment of the pocket must be an advantage to enable capture of the oxyanion, even when the extracellular concentration is low. Several residues are likely to be involved in ligand binding, attracting, accommodating and delivering the oxyanion to the membrane component of the transporter system, TupB. The TTTS motif is in lobe A, with the serine pointing towards the metal binding site. Opposite to these are R118, T124 and D170, also likely involved in oxyanion interaction (Fig. 7). These residues are highly conserved among other TupA proteins from *Desulfovibrio* species but also extending to proteobacteria, Green Non Sulfur bacteria and even to Gram-positive bacteria such as Firmicutes. When searching for similar sequences with the TTTS motif and excluding the *Desulfovibrio* genus, over 500 sequences were found with more than 44% identity.

Moreover, one chloride anion, arising from the crystallization conditions, was found at 3.8 Å from R118, indicating the propensity of the pocket to attract negatively charged ions. Although phosphate buffer was used during protein purification this ion is not occupying the WO_4_^2−^ binding site, in agreement with what is already known for this type of transporters and with our previous experimental data where we showed that *Da*G20 TupA is highly specific for WO_4_^2−^ /MoO_4_^2−^ and not for other oxyanions as SO_4_^2−^, PO_4_^3−^ and ClO_4_^−^^31^. To examine the importance of R118 in ligand binding, site-directed mutagenesis has been carried out, as described in the following section.

### The relevance of arginine 118 in the oxyanion binding

Using ITC, we reported the binding affinity of *Da*G20 TupA for tungstate and molybdate (K_d_ of 6.30 ± 0.02 pM and 6.1 ± 0.9 nM, respectively)^31^. The described dissociation constants agree with what has been observed for the putative TupA Cj1540 from *C. jejuni*, with this protein binding more tightly tungstate (K_D_ 1.0 ± 0.2 pM) than molybdate (K_D_ 50 ± 10 nM)^26^.

**Figure 8:**
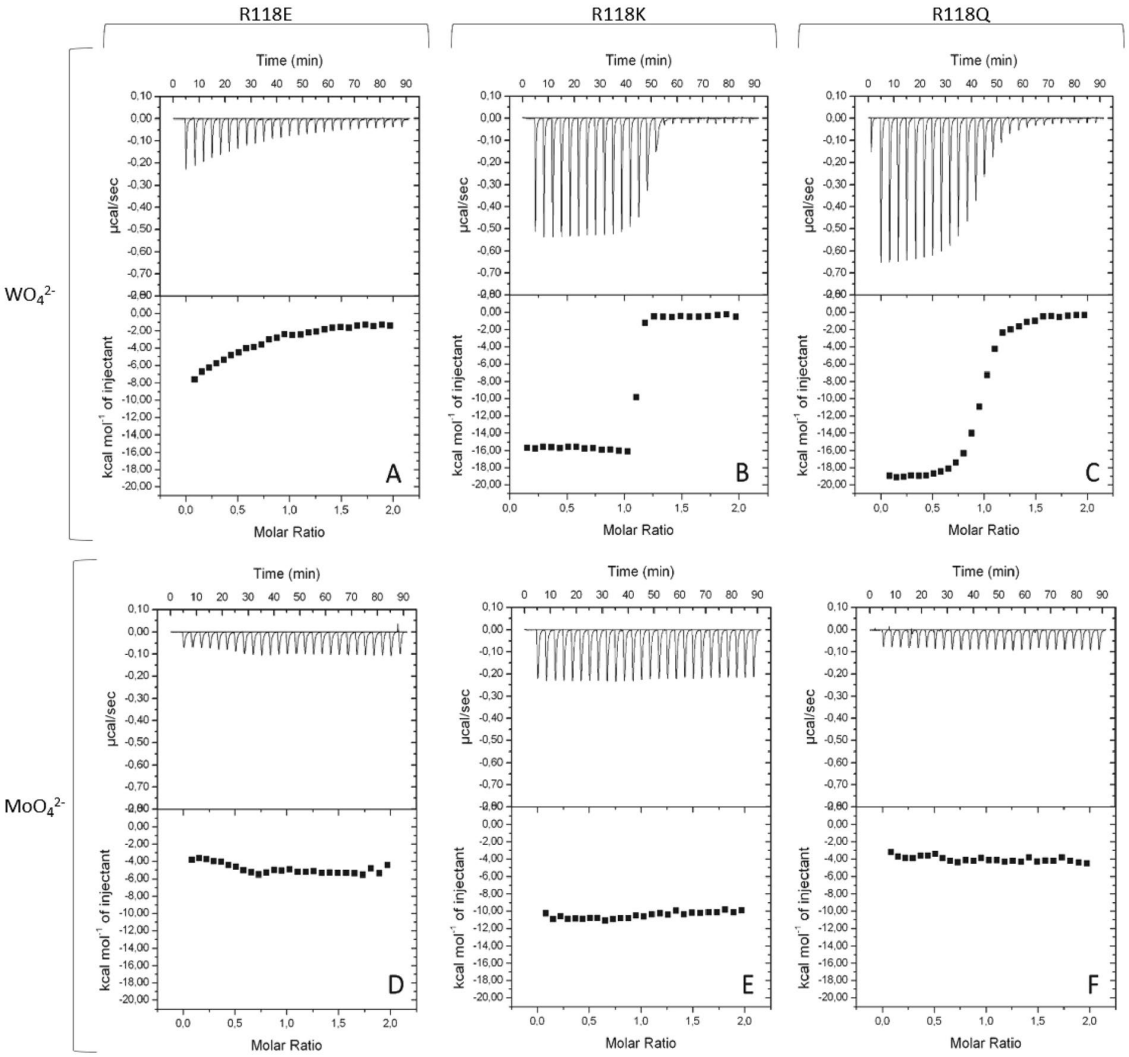
Isothermal titration calorimetry of ligand binding to TupA variants. 10 µM of the variant R118E (**A,D**), R118K (**B,E**) or R118Q (**C,F**) was titrated with injections of 100 µM of WO_4_ (**A,B,C**) and 100 µM of MoO_4_ (**D,E,F**). Data were fitted with ORIGIN software. The raw ITC data are shown in the top graphs.

The same methodology was used to attend the ability of the R118E/Q/K variants for oxyanion binding (see Fig. 8 and Table 2). The results show that substitution of the positive side chain by a carboxylic acid (R118E variant) prevents the binding to both molybdate and tungstate, confirming the relevance of this conserved residue. When R118 is replaced by a glutamine (R118Q variant), the protein binds tungstate with much less affinity than the wild-type (K_D_ of 90 ± 50 nM), also losing the ability to bind molybdate. However, if the arginine is replaced by a lysine, the results are remarkably different. Analysis of the ITC thermograms of R118K titrated with tungstate shows a very steep binding curve that indicates a strong interaction. The obtained curve hampers the determination of K_D_, extremely smaller as the determined for the wild-type. This problem was also observed before for the *DaG20* TupA-tungstate binding but was overcome by competition studies using molybdate. However, R118K totally lost the affinity to bind molybdate and such binding competition strategy cannot be adopted. Nevertheless, the results clearly show that R118K variant has an extremely high affinity and selectivity for tungstate.

**Table 2:** ITC analysis of tungstate binding to TupA variants at 30 °C. In each case 10 µM protein was used for the titrations.

Variant	n	K_A_ (M^−1^)	K_D_ (nM)	ΔH (kcal mol^−1^)
TupA^31^	0.845 ± 0.003	(1600 ± 6) × 10^8^	(6.30 ± 0.02) × 10^−3^	−14.60 ± 0.04
R118K	1.080 ± 0.003	(6 ± 2) × 10^8^	1.8 ± 0.8	−15.8 ± 0.1
R118Q	0.950 ± 0.003	(0.111 ± 0.006) × 10^8^	90 ± 50	−19.40 ± 0.08
R118E	No binding			

n = measured stoichiometry of binding.

This contrasts with what has been observed for *E. acidaminophilum* where the authors reported that the R/K mutant strongly diminishes the binding of tungstate^16, 32^.

## Conclusions

Tungstate is a widely used heavy metal which is raising environmental concerns due to bioaccumulation in soils and water. Currently, tungsten detection requires expensive equipment (such as ICP-mass spectrometry) with only very few reports on new detection methodologies that can be applied directly on the field. A promising research field is the use of enzyme biosensors for determining toxic compounds since the associated analytical systems are simple, rapid and selective. Insertion of tungsten (and molybdenum) in bacterial enzymes is crucial for its activity and requires an uptake system, usually in the form of oxyanion. The organisms developed an efficient biological system, an ABC-type transporter that allows the metal uptake as well as differentiation between the two similar elements, W and Mo. Three classes of ABC transporting systems are described in the literature, in particular ModABC, WtpABC and TupABC.

In this work, we characterized structurally and biochemically the oxyanion coordination and selectivity of the wild-type *Da*G20TupA and three R118 variants. X-ray crystallography and SAXS, reveals that TupA architecture has a common feature with other substrate binding proteins, which is the existence of two separate lobes. This characteristic may improve the interaction of the periplasmic component with the dimeric transmembrane component (TupB), but also provide the structural flexibility that allows TupA to switch between a loose or compact conformation in the absence or presence of the oxyanion, respectively.

The ITC results clearly indicate the relevance of R118 in the oxyanion coordination where three mutants lost the ability to bind molybdate. Curiously, for the R118K variant, the residue substitution increases the selectivity of the protein towards tungstate with high affinity, depleting its ability to bind molybdate.

Due to the conformational changes of TupA upon ligand binding and the high affinity and selectivity of the genetically modified variant R118K, this protein can be considered for biotechnology applications. An alkaline phosphatase based biosensor was recently described to detect W in aqueous media by Alvarado-Gámez and co-workers. The detection relies on the fact that tungstate affects the activity of the immobilized enzyme. However, this method is highly compromised by the presence of other elements like selenium, iron, calcium or aluminum, that even at low concentration interfere with the heavy metal detection^33^. The remarkable advantage of using TupA_R118K as the bioreceptor component of a sensor is that it’s activity is not affected by the presence of other similar ions like molybdate, sulfate, phosphate or perchlorate, allowing selective detection. Combining different techniques, we could better understand the mode of binding of tungstate in TupABC system, paving the way to the development of a new trend in W detection.

## Methods

### DNA Cloning and Site-directed Mutagenesis

The pet46-tupA expression vector^31^ containing the *tupA* gene (locus tag Dde_0234) was used as a template to perform site-directed mutagenesis of the R118 residue. Primers listed in Table 3 were designed to construct the pET46-tupA_R118K, pET46-tupA_R118E and pET46-tupA_R118Q variants using the Site-Directed Mutagenesis Kit (QuikChange^*®*^, Stratagene) following the manual kit instructions. XL1-Blue supercompetent cells were transformed with each expression vector and plasmids were isolated from a unique colony using the NZY-Tech Miniprep kit (NZY-Tech, Lisboa, Portugal). The variants constructs were confirmed by DNA sequencing using an ABI3700 DNA analyzer (Perkin/Elmer/Applied Biosystems, STABvida, Caparica, Portugal). The sequences were aligned and analyzed using the online tool BLASTp^34^ and ClustalW^35^.

**Table 3:** Primers used to mutate the R118 residue.

Primer	Sense	Sequence	Variant
fTupA_R118K	forward	CCGTATTTGTAAGC**AAG**GGCGACAACTCGG	R118K
rTupA_R118K	reverse	CCGAGTTGTCGCC**CTT**GCTTACAAATACGG	
fTupA_R118E	forward	CCGTATTTGTAAGC**GAG**GGCGACAACTCGG	R118E
rTupA_R118E	reverse	CCGAGTTGTCGCC**CTC**GCTTACAAATACGG	
fTupA_R118Q	forward	CCGTATTTGTAAGC**CAG**GGCGACAACTCGG	R118Q
rTupA_R118Q	reverse	CCGAGTTGTCGCC**CTG**GCTTACAAATACGG	

### Expression of TupA variants

The heterologous expression conditions of TupA previously optimized^31^ were tested for the three variants. These conditions yield a high percentage of protein in the insoluble fraction (data not shown). To overcome this problem, several parameters were tested, such as IPTG concentration (0.1, 0.3, 0.5, 0.8 and 1 mM) and different strains of expression hosts (Rosetta 2(DE3)pLysS, Origami (DE3) and Tuner (DE3), from Merck Millipore). The best results were obtained using *E. coli* Rosetta 2 and Origami cells for pET46-tupA_R118K and pET46-tupA_R118E, respectively. In both cases, the cells were transformed and cultured in sterile Luria-Bertani medium supplemented with ampicillin (100 µg/mL) at 180 rpm and 37 °C. When OD_600_ reached 0.6, the protein expression was induced using 1 mM IPTG and cells were grown for 16 hours at 19 °C. For the pET46-tupA_R118Q plasmid, *E. coli* Tuner cells were transformed and the expression performed as previously described but using 0.3 mM of ITPG during the induction period.

### Protein isolation protocol

After protein expression, the cells were harvested at 5000 × g for 20 min and the pellet was resuspended in a ratio of 2 g cells/mL in 50 mM sodium phosphate buffer (pH 8.0) containing 500 mM NaCl, 5 mM DNase and 1 tablet/L of Protease Inhibitor Cocktail - EDTA (Sigma-Aldrich). The cells were disrupted by sonication and the solution clarified by centrifugation (13000 × g for 30 min). The soluble fraction was filtered through a 0.45 µm membrane.

Protein purification was performed in a one-step protocol using an immobilized-metal affinity chromatography (IMAC), His GraviTrap (GE Healthcare) following the manufacturer’s instructions. Target proteins were eluted using 50 mM sodium phosphate buffer (pH 8.0) containing 500 mM NaCl and 250 mM imidazole. The fractions were analyzed by 10% tris-tricine/polyacrylamide gel electrophoresis stained with Coomassie blue. Fractions containing TupA_R118K, TupA_R118E and TupA_R118Q were dialyzed against 5 mM Tris-HCl pH 7.6. All of the steps were performed at 4 °C.

### Isothermal Titration Calorimetry

ITC experiments were performed as described previously^31^ using a VP-ITC calorimeter (MicroCal GE Healthcare). Prior to experiments, the protein was extensively dialyzed against the reaction buffer (5 mM Tris-HCl, pH 7.5, prepared with Milli-Q dH2O). The reaction cell containing 10 µM of protein was equilibrated at 30 °C, titrated with sodium tungstate or molybdate (20 or 23 injections of 10 µl of a 100 µM oxyanion solution) and the heat response recorded. After subtraction of the baseline, the integrated heats were fitted to the single binding site model using the ORIGIN software package supplied with the calorimeter to derive, n, Ka and ΔH values.

### Urea-polyacrylamide gel electrophoresis

The stability of TupA after ligand binding (tungstate and molybdate) was analyzed by urea gel electrophoresis using the Novex 6% tris(hydroxymethyl) aminomethane (Tris)-borate (TBE)-urea minigels and a XCell SureLock™ Mini-Cell (Invitrogen), as previously described by Mehtab *et al*.^36^. The protein (5 µL, 50 µM) was mixed with 5 µL of 2× Novex sample buffer (without EDTA). The electrophoresis was carried out for 150 min at 180 V and 40 mA. The protein bands were examined using Coomassie blue staining. In order to avoid the metal chelation, EDTA was removed from the electrophoresis solutions.

### Crystallization of TupA

Crystallization experiments were performed using the hanging drop vapor-diffusion method, adding 2 µL of TupA (7.5 mg/mL) to 1 µL of precipitating solution containing 0.2 M magnesium chloride, 0.1 M HEPES (pH 7.5) and 30% (w/v) polyethylene glycol 3350. Colorless crystals with maximum dimensions of 0.3 × 0.15 × 0.06 mm^3^ appeared within four days. For further details, see Otrelo-Cardoso *et al*.^31^.

To obtain a crystal structure of the holo form of TupA (MoO_4_^2−^/WO_4_^2−^ -TupA) soaking and co-crystallization experiments were performed. For the soaking experiments, crystals obtained in the described condition were stabilized by adding a harvesting buffer solution containing 32% (w/v) PEG 3350. The crystals were then incubated with a 5, 10 or 20-fold excess of ligand (prepared in 0.2 M magnesium chloride, 0.1 M HEPES pH 7.5 and 32% (w/v) PEG 3350) for 10 min to 24 hours. Macroscopically, the crystals were not damaged during the soaking and were afterward flash frozen using the mentioned cryo-protectant. Although more than 120 crystals were tested, almost all had poor to non-existing diffraction.

### Data Collection, structure determination and refinement

Crystals were flash-cooled in liquid nitrogen using Paratone oil as a cryoprotectant and maintained at 100 K under a stream of gaseous nitrogen during data collection. A complete dataset was collected at beamline ID23-1 at the European Synchrotron Radiation Facility (ESRF, Grenoble, France). The crystals diffract beyond 1.40 Å resolution at a wavelength of 0.954 Å and belong to space group P12_1_1. Data was processed with the XDS^37^ package and AIMLESS^38^ from the CCP4 program package v. 6.3.0 (Collaborative Computational Project, Number 4, 1994)^39^. The data collection and processing statistics are presented in Table 4.

**Table 4:** Data collection, processing and structure refinement statistics for TupA crystal. Values in parentheses correspond to the highest resolution shell.

Data collection, processing and structure refinement statistics	
X-ray source	ID23-1 (ESRF, Grenoble)
Detector	PILATUS 6M-F
Wavelength (Å)	0.954
Unit-cell parameters (Å, °)	a = 52.32; b = 42.53; c = 54.75; β = 95.45
Space group	P12_1_1
Molecules per AU^#^	1
Matthews coefficient (Å^3^, Da)	2.09
Mosaicity (°)	0.22
Resolution range (Å)	42.53–1.40 (1.42–1.40)
<I/σI>	12.8 (2.4)
R_merge_ (%)	3.4 (29.7)
R_pim_ (%)	3.3 (28.8)
R_meas_ (%)	4.8 (41.4)
Multiplicity	2.9 (2.9)
No. of observed reflections	135886 (6650)
No. of unique reflections	46519 (2277)
Completeness (%)	98.3 (98.5)
R_free_^*^ (%)	21.7%
R_factor_ (%)	17.6%
Number of water molecules	332
Other heteroatoms	1 sodium, and 2 chloride
Average B factor for all atoms (Å^2^)	23.67
RMSD from ideal geometry	
Bond lenghts (Å)	0.022
Bond angles (°)	2.017

^#^AU: asymmetric unit. ^*^R_free_ was calculated for 5.1% of the reflections randomly chosen from the data set.

Structure determination was carried out by molecular replacement using PHASER^40^ and several molecular models were selected according to sequence alignment homologies, namely: a conserved functionally unknown protein from *Vibrio parahaemolyticus* serotype O3:K6 (PBD code 3muq) and the *Gs*TupA (PDB code 3lr1), after omitting all the cofactors and solvent molecules. The MR solution could only be obtained when the two models were superposed and small domains of the protein were used separately: Domain I, including the first 81 residues; Domain II comprising residues 82 to 188; and finally, Domain III with residues 189 to 236. After structure solution, Buccaneer was used for the automated model building^41^ and REFMAC 5 for restrained refinement^42^. The water molecules were automatically added by REFMAC 5 and manually inspected in Coot^43^. Geometrical validation and model improvement was carried out using PDB_REDO^44^ and the final values of 0.176 and 0.217 for R and R-free factors were obtained, respectively. The Ramachandran plot has 97.15% of the residues in the preferred regions, without any outliers. Mean bond angle and bond length deviations from ideal values and other refinement statistics are presented in Table 4. The deposited model contains 250 protein residues, 332 water molecules, two chlorides and one sodium ion. During the electron density inspection, two mutated residues were found: R107K and S138P. These mutations are likely to come from the cloning strategy but, since are located far from the tungstate binding pocket were considered irrelevant for structure analysis.

### Small-angle X-ray scattering, data collection and analysis

SAXS data was collected at EMBL P12 beamline, DESY, Hamburg, Germany and at EMBL BM29 beamline, ESRF, Grenoble, France with protein concentration ranges of 12–0.5 mg/ml. Data was cropped from the first point of the Guinier region until the end of the useful range defined by SHANUM^45^. High and low concentration curves were merged to counter concentration effects such as interparticle interference using the program PRIMUS from the ATSAS package^46^. GNOM^47^ was used to obtain the *p*(*r*) and determine the corresponding and values. BUNCH^29^ and SREFLEX^30^ were used to generate and refine high-resolution hybrid models using the crystallographic structure reported here as the starting point. The scattering curves from the high-resolution models were calculated using CRYSOL^48^.

### Data Availability

Coordinates and observed structure factor amplitudes of *DaG20* TupA have been deposited in the Protein Data Bank under the accession code 5my5. The collected SAXS data and the generated high-resolution hybrid models have been deposited and are available at SASBDB (entries: SASDBD9, SASDBE9, SASDBF9, SASDBG9 and SASDBH9)^49^.
